# Keep Your Friendships in Repair

**Published:** 1918-12-28

**Authors:** 


### THE YEARS THAT LIE AHEAD.

### Keep Your Friendships in Repair.

Many brave men, says Horace, lived before Agamemnon; and we may be sure that many wise men lived before Solomon; and many men as brave have lived since Agamemnon succumbed to the fatal hatchet of Clytemnestra; many men as wise have lived since Solomon in all his glory received the Queen of Sheba. In all this witenagemot it would be hard to find a wiser man, prejudiced and irascible though he was, than that tender-hearted sage, Dr. Samuel Johnson. It would be hard to meet with any situation in life for which an appropriate precept, an apt quotation, a fitting admonition cannot be found in the table-talk of Dr. Johnson; and of all his wise precepts none is wiser, none is more generally applicable, none is less hackneyed, than that which bids us keep our friendships in repair.

The admonition may be taken in two ways, it may have two meanings, and both are judicious and worthy to be followed. It may mean that we should take pains to maintain, consolidate, and renew the friendships we have already formed, or it may be taken to mean that we should, by making new friends, repair the breaches that Time inevitably makes in our friendships by death, by removal, and by other modes of severance.

A man should keep his friendships in repair.

"Friends!" says the Chinese proverb, "Has the word a plural?" That depends, perhaps, on the sense in which we use the word. There is one kind of friend of which no man can expect to possess more than one specimen—the intimate friend who knows all our affairs, to whom we can turn in sorrow and ill-fortune for consolation, in joy and good fortune for congratulation, in difficulty for judicious counsel, and in all circumstances for ready understanding, sympathy, and affection. This is the friend of whom Polonius speaks—"the friends thou hast, and their adoption tried, grapple them to thy soul with hooks of steel," and happy is the man who can count upon but one. But beyond this other self we all have friends, less intimate, but still close, dear, and good, ready to befriend and to be befriended, who are glad to see us, and whom we are glad to see, to whom we give pleasure while adding to our own, with whom the interchange of good offices and social amenities knits closer the bonds of social life, broadens the sympathies, widens the current of our lives, and increases the joys of existence. A man should keep such friendships in repair.

To keep our friendships in repair is a social duty. A house divided against itself cannot stand. A society divided into antagonistic cliques, a nation cursed by a war of classes, is rotten, frail, and on the way to destruction. Before the war that is now so happily concluded, there existed in this country a smouldering hostility between class and class, an hostility that certain ill-advised, though doubtless well-meaning, agitators were doing their best to fan into a devouring flame. A more pernicious and detestable propagandism it would be difficult to conceive, and it is a curious commentary upon the inconsistency and illogicality of mankind that the arch-agitator, who taught that virtue and merit are in inverse proportion to social position; that the most perfect human being is the day labourer, and the most depraved specimens of humanity are the dukes, the man who was stirring up a class war and preaching the morality of confiscation and strife, is a pious confessing Christian, committed to the doctrine of peace on earth and goodwill amongst men!

> There is some soul of goodness in things evil
> Would men observingly distil it out.

and even this terrible war, which has desolated millions of homes and brought misery to tens of millions, has yet not been wholly without benefit. In this country, at any rate, it has brought the different classes together, has taught them to know one another, and has counteracted by practical experience much of the pernicious doctrine of class-hatred that was being so furiously instilled before the war. The classes have come together, have combined their efforts for a common purpose, have lived in more intimate association than ever before, and have learned to appreciate each other's good qualities. The day labourer and the shop assistant have displayed heroism no less conspicuous and astonishing than that of the duke and the earl; the duke and the earl have displayed a patient and uncomplaining endurance of hardship, misery, and wounds, no less admirable than that of the chimney-sweep and the man before the mast. All classes have learned to respect and like one another, and in very many cases the most incongruous associates have become friends. These are the friendships that should be kept in repair.

Another soul of goodness that we may distil out from the gigantic evil of the great war is the utter discrediting of the professional politician. He has been weighed in the balances and found wanting. He is a bladder that has been pricked and has collapsed. The hollowness and emptiness of his calls to internecine strife between class and class, between capital and labour, have been exposed. They were never more than a means by which he might raise himself to power and salary, and they are now pretty well known to be no more. Let us hope that the sordid auction of bribes to the electorate, in which each party strive to outbid the other in promises that were never kept, is now closed. It seems manifest that if two men are pulling on a rope they will produce more effect if they both pull together in the same direction than if they face one another and pull opposite ways; but this is what capital and labour have been doing for years. Why cannot they pull together? Their main interests are identical, if they would but see it. The subordinate interests, which are diverse, can be adjusted if there

**Keep Your Friendships in Repair**—(*continued*).

is good-will, and if the friendships made during the war are kept in repair. The truth is not sufficiently recognised that *every commercial transaction is a failure unless both parties are satisfied.* Let capital and labour meet and discuss their affairs with this truth in their minds, and with the determination that both parties shall be satisfied. Let them meet, not as enemies, desiring to get the better of each other, but as friends desiring to do the best for themselves, for each other, and for their country. If they neglect the last two, they will certainly fail, in the long run, to benefit the first. The old feudal relationship had too much of patronage and condescension on one side, and too much of subservience on the other; but at least it was a relation of friendship, it was kept in repair, and it endured for many hundred years. The new polity must take a new shape, but if it is to endure, if it is to be beneficial to all, if it is to lead to satisfaction and contentment, it must be based not upon antagonism, not upon hostility, but upon friendship, upon friendship that is perpetually kept in repair by friendly intercourse and friendly offices. So, and no otherwise, shall there be peace on earth and good-will amongst men.

What the opposite course leads to we see in Russia. It leads to anarchy, murder, terror, and starvation.

